# Effect of natural honey from Ilam and metformin for improving glycemic control in streptozotocin-induced diabetic rats 

**Published:** 2012

**Authors:** Ozra Nasrolahi, Reza Heidari, Fatima Rahmani, Farah Farokhi

**Affiliations:** 1 *Department of Biology, Faculty of Science, Urmia University, Urmia, I.R. Iran*

**Keywords:** Diabetes, Honey, Metformin, Streptozotocin (STZ)

## Abstract

**Objective(s): **Diabetes mellitus is a public health problem and one of the five leading causes of death globally. In the present study, the effect of Metformin with natural honey was investigated on glycemia in the Streptozotocin-induced diabetic rats.

**Materials and Methods: **Thirty Wistar male rats were randomly divided into six groups including C: non diabetic rats received distilled water, CH: non diabetic rats received honey, CD: diabetic rats administered with distilled water, DM: Metformin treated diabetic rats, DH: honey treated diabetic rats, and DMH: diabetic rats treated with a combination of Metformin and natural honey. Diabetes was induced by a single dose of Streptozotocin (65 mg/kg; i.p.). The animals were treated by oral gavage once daily for four weeks. At the end of the treatment period, the animals were sacrificed and their blood samples collected. Amount of glucose, triglyceride (TG), total cholesterol (TC), HDL cholesterol, LDL cholesterol, VLDL cholesterol, total bilirubin, and albumin were determined in serum.

**Results: **Group CD: showed hyperglycemia (252.2±4.1 mg/dl), while level of blood glucose was significantly (p<0.01) reduced in groups DH (124.2±2.7 mg/dl), DM (108.0±3.4 mg/dl), and DMH (115.4±2.1 mg/dl). Honey in combination with Metformin significantly (p<0.01) reduced level of bilirubin but Metformin alone did not reduce bilirubin. Honey alone and in combination with Metformin also significantly reduced triglycerides, total cholesterol, LDL, VLDL and increased HDL, but Metformin did not reduced triglycerides and increased HDL.

**Conclusion: **The results of the present study demonstrated that consuming natural honey with Metformin improves glycemic control and is more useful than consuming Metformin alone. The higher therapeutic effect of Ilam honey on lipid abnormalities than Tualang honey was also evident.

## Introduction

Diabetes is a metabolic disease recognized by chronic hyperglycemia. This disease is the result of deficiency in producing insulin and influences carbohydrates, proteins, and lipids metabolism (Nathan, 1993 ▶). The prevalence of diabetes is increasing in both industrialized and developing countries (Kar et al., 1999 ▶). Increase in the incidence of diabetes, has posed this disease as a serious factor in a health threat in the 21st century (Cheng, 2005 ▶). 

In the past three decades, there has been a significant progress in diabetes treatment, but the health outcomes of patients are still the most appropriate level of treatment in both industrialized and developing countries (Murugesan et al., 2009 ▶). Hyperglycemia increases glucose metabolism, cholesterol level, and lipid peroxidation. It also increases oxidative stress causing production of free radicals which in turn will lead to physiological disorders and structural damages in different tissues of the body including brain, kidney, liver, retina, and cardiovascular system (Erejuwa et al. 2011a ▶). Diabetes mellitus is frequently associated with impaired lipid metabolism such as elevated cholesterol and triglycerides (TG) and abnormalities in serum lipoproteins (Erejuwa et al., 2012a ▶).Impairment in synthesis or metabolism of lipoproteins is an impending atherogenic risk factor in diabetes and microalbuminuria is one of the clinical manifestations of diabetic nephropathy (Erejuwa, 2011b ▶). 

Studies have shown that diabetes could not be effectively controlled with only one medication (Erejuwa et al., 2011b ▶). Furthermore, the available drugs have undesirable side effects such as hypoglycemia, weight gain, and limitations in preventing destruction of the pancreas and diabetes complications associated with oxidative stress (Erejuwa et al., 2011b ▶). Honey has been used in traditional medicine to treat wounds, burns, gastrointestinal disorders, asthma, and cataracts around the world (Pérez et al., 2006 ▶; Miguel Alvarez-Suarez et al., 2010) and has recently become a subject of renewed research interest in the last few years (Erejuwa et al., 2012b ▶). 

Natural honey has many medicinal effects such as antibacterial, hepatoprotective, hypoglycemic, reproductive, antihypertensive, and amelioration of oxidative stress in kidneys and pancreas of STZ-induced diabetic rats (Erejuwa et al., 2012b ▶; Erejuwa et al., 2010a ▶; Erejuwa et al., 2010b ▶). Honey is a supersaturated sugar solution of fructose and glucose (Rosa et al., 2011 ▶). Besides carbohydrates, it contains a wide range of compounds such as convention minerals, proteins, vitamins, organic acids, enzymes, and antioxidants such as catalase, peroxidase, alkaloids, polyphenols, and flavonoids (Gheldof et al., 2002 ▶). Studies have shown that the antioxidant capacity of honey is dependent on the concentration of phenolic groups (Gheldof and Engeseth, 2002 ▶). Moreover, type of antioxidant is largely dependent on floral sources, flowers, geographical area, total phenolic content, water proportion, and color (Rosa et al., 2011 ▶; Erejuwa et al., 2011c ▶).

Metformin is an oral hypoglycemic agent and a drug for regulating blood sugar. Its main task is to increase insulin sensitivity in liver and facilitate the transport of glucose in hyperglycemia and insulin resistance (Soto et al., 2008 ▶). Induction of diabetes is associated with decreased body weight in rats. Drugs such as Metformin and Glibenclamide control blood sugar level but do not prevent weight loss in diabetic rats (Erejuwa et al., 2011b ▶). Ilam natural honey has high antioxidant properties due to unique and high diversity of medicinal plants in Ilam forests. The result of physicochemical tests conducted on Ilam honey shows that this honey is completely in accordance with national and international standards. Virgin term is used by European laws to show that honey is natural (Europe union, 2002b). One of the limitations of this law in European Union to introduce honey as a virgin honey is humidity up to 18 percent and the amount of HMF up to 25 mg / kg (Feás et al., 2010 ▶). On this basis, Ilam honey duo to the presence of 17.5% moisture and HMF about 3.40 mg/kg can be labeled as virgin honey. The radical scavenging activity was performed by DPPH assay (% inhibition) and 57.25 mg/ml was resulted. The antioxidant activity was performed by FRAP assay and 328.32 μM Fe (II) was assessed. The results show that Ilam honey has higher antioxidant properties compared with Tualang honey (Mohamed et al., 2010 ▶; Erejuwa et al., 2011 ▶). However, no research has been conducted on beneficial effects of Iranian honey on diabetes. In the present study, we aimed to study the effect of Ilam natural honey as an effective antioxidant and Metformin on glycemic control in STZ-induced diabetic rats. Our results would be beneficial for further investigations on application of this type of natural honey in diabetes treatment.

## Materials and Methods

**Animals**

The current study was conducted on 30 healthy male Wistar rats weighing 200±20 g. All animals were kept in standard conditions with constant 12h light/12h dark cycle at temperature of 25±2 °C. The rats were fed with standard food and tap water *ad libitum.*

Rats were purchased from Pasteur Institute, Tehran, Iran. The animals were handled in accordance with the Institutional Guidelines for the Care and Use of Animals for Experimental Purposes. The animals were acclimatized to the animal room condition for at least one week prior to the experiment.

**Preparation of natural honey and Metformin**

Honey was obtained from forests of Ilam province and its natural source was confirmed by the Agricultural Research Center of Ilam. Physicochemical properties of honey were measured according to harmonized methods of the International Honey Commission (Bogdanov, 1999) and National Iranian Standard No. 92 (honey - Specifications and test methods). This honey contained 0.986% of total reducing sugar including fructose (35.56 %), glucose (30.04%) and fructose/glucose ratio (0.99), sucrose (1.14%), water (17.5%), hydroxymethyl furfural (HMF) (3.40 mg/kg), radical scavenging activity (57.25% inhibition) and anti-oxidant activity of 328.32 μM Fe (II). Honey (1.0 g/kg body weight) was freshly dissolved with distilled water just before each administration. Metformin (100 mg/kg body weight) was dissolved in distilled water before administration. 

**Induction of diabetes**

After an overnight fast, diabetes was induced by intraperitoneal administration of Streptozotocin (65 mg/kg BW dissolved in normal saline). Three days after Streptozotocin injection, development of diabetes was confirmed by measuring glucose level in fasting blood samples taken from tail vein. Glucose measurement was performed with an Accu-Chek glucometer (Roche, Germany). Rats with blood glucose concentration of 220 mg/dl or higher were considered diabetic and included in the study. 

**Animal treatment**

The current study was conducted on healthy adult male Wistar rats weighing 200±20 g. Rats were weighed before induction of diabetes. The animals were randomly allocated to six groups and treated as follows: Non-diabetic control rats received distilled water (C), non-diabetic control rats received natural honey (CH), diabetic control rats received distilled water (CD), diabetic rats administered with Metformin (DM), diabetic rats administered with natural honey (DH) and diabetic rats administered with Metformin and natural honey (DMH). Metformin (100 mg/kg BW), natural honey (1.0 g/kg BW), and distilled water were administered orally (by intragastric tube) once daily for 4 weeks. At the end of the treatment period, the rats were weighed again, fasted for at least 16 hours and sacrificed by decapitation. Blood samples were collected in centrifuge tubes without anticoagulants and allowed to clot. The clotted blood was then centrifuged at 3000 x g for 20 minute. Serum was separated and then quickly stored at -80 °C for biochemical analyses. 

**Biochemical analyses**

Amount of glucose, TG, LDL, VLDL, HDL, total bilirubin, and albumin in serum were determined using commercially available kits. Measurements were carried out in 14000 auto analyzer (Toshiba, Japan) using manual colorimetric method.

**Statistical analysis**

All values were expressed as mean± SEM. The differences were compared using one way analysis of variance (ANOVA) followed by Tukey tests and p<0.01 were considered statistically significant.

## Results

**Body weight**

**Figure 1:**
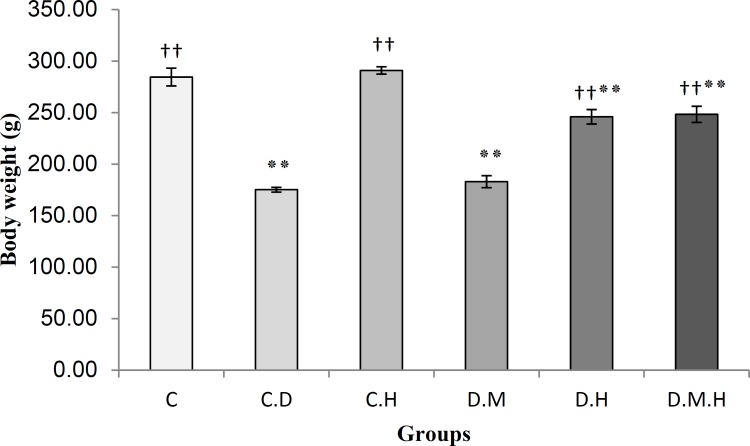
ffects of natural honey, Metformin, or their combinations on body weight in STZ-induced diabetic rats. C (Non- diabetic control), CH (Non- diabetic+ Honey), CD (Diabetic control), DH (Diabetic+ Honey), DM (Diabetic+ Metformin) and DMH (Diabetic+ Metformin+ Honey). Data are expressed as mean± SEM. Each group consisted of five animals. Values are statistically significant at *p<0.05, **p<0.01 compared with C; †p<0.05, ††p<0.01 compared with CD

Figure 1 summarizes the results of the body weight in different groups of rats. There was no significant difference in body weight among the groups before the commencement of the study (results not shown). The diabetic control rats had significantly (p<0.01) reduced body weight compared with non-diabetic control rats. The diabetic groups that received natural honey or Metformin in combination with honey showed significant (p<0.01) improvement in body weight compared with diabetic control rats. However, Metformin alone did not lead to improvement in diabetic rats.

**Serum glucose, bilirubin and albumin**

Table 1 summarizes the results of serum glucose, bilirubin, and albumin in different groups of animals at the end of the four-week treatment period. The serum glucose concentration of the diabetic control rats was significantly higher (252.2±4.1 mg/dl) than those of the non-diabetic control rats (72.4±2.2 mg/dl). Treatment with Metformin or honey significantly decreased the glucose level (108.0±3.4 or 124.2±2.7 mg/dl, respectively) in diabetic rats. Combination of Metformin and honey reduced the glucose concentrations (115.4±2.1 mg/dl) in diabetic rats. No significant effect of honey in non-diabetic rats was observed (Table 1).

Serum level of bilirubin was significantly (p<0.01) increased in the diabetic control rats compared with non-diabetic rats. Serum levels of bilirubin remained elevated in the diabetic rats treated with Metformin. However, Metformin combined with natural honey significantly (p<0.01) decreased serum bilirubin in diabetic rats compared with diabetic control rats. The serum albumin level did not change in different groups (Table 1). 

Table 2 depicts the average data of glucose, bilirubin, and albumin in different groups of animals. Averages are shown in term of best case with letters A, B, C, and D, respectively.

**Serum triglycerides, total cholesterol, HDL, LDL, and VLDL cholesterol**

Table 3 shows the serum levels of triglycerides, total cholesterol, HDL cholesterol, LDL cholesterol, and VLDL cholesterol of control and STZ-induced diabetic rats. Interestingly, increased levels of TG, VLDL cholesterol, total cholesterol, and LDL cholesterol and decreased levels of HDL cholesterol were observed in diabetic control rats compared with non-diabetic rats. Administration of natural honey or natural honey with Metformin significantly (p<0.01) decreased the levels of TG and VLDL cholesterol in diabetic rats compared with diabetic control rats and diabetic rats treated with Metformin alone. Besides, natural honey and combination of Metformin and natural honey increased the levels of HDL cholesterol compared with Metformin alone in diabetic rats. Natural honey, Metformin and combination of Metformin and natural honey significantly (p<0.01) decreased the levels of TC and LDL cholesterol in diabetic rats compared with diabetic control rats. Table 4 depicts the average data of the triglycerides, total cholesterol, HDL cholesterol, LDL cholesterol and VLDL cholesterol in different groups of animals. Averages are shown in term of best case with letters A, B, C and D, respectively.

**Table 1:** Effects of natural honey, Metformin, and their combinations on serum glucose, bilirubin and albumin ratio in the serum of STZ-induced diabetic rats

**Group**	**Serum glucose(mg/dl)**	**Bilirubin (mg/dl)**	**Albumin (mg/dl)**
**None-diabetic Control (C)**	72.4±2.2††	0.31±0.03††	3.62±0.21
**Non-diabetic+ Honey (CH)**	78.2±3.3†	0.35±0.02††	4.24±0.19
**Diabetic Control (CD)**	252.2± 4.1٭٭	0.85±0.02٭	4.2±0.1
**Diabetic+ Honey (DH)**	124.2±2.7††٭٭	0.78±0.02٭٭	4.48±0.15
**Diabetic+ Metformin (DM)**	108.0±3.4 ††٭٭	0.76±0.03٭٭	3.5±0.37
**Diabetic+ Metformin+ Honey (DMH) **	115.4±2.1 ††٭٭	0.69±0.04٭٭††	4.18±0.13

Data are expressed as mean±SEM. Each group consisted of 5 animals. Values are statistically significant at :

* p<0.05,

** p<0.01 compared with non-diabetic control;

† p<0.05,

†† p<0.01 compared with diabetic control.

**Table 2:** Comparison in terms of p< 0.05 for glucose, bilirubin and albumin. Common letters indicate non-significant differences between groups

**Group**	**Serum glucose (mg/dl)**	**Bilirubin (mg/dl)**	**Albumin (mg/dl)**
**None-diabetic Control (C)**	A	A	AB
**Non-diabetic+ Honey (CH)**	A	A	AB
**Diabetic Control (CD)**	D	C	AB
**Diabetic+ Honey (DH)**	C	BC	B
**Diabetic+ Metformin (DM)**	B	BC	A
**Diabetic+ Metformin+ Honey (DMH)**	BC	B	AB

**Table 3:** Effects of honey, Metformin, or their combinations on triglycerides (TG), total cholesterol (TC), high density lipoprotein (HDL) cholesterol, low density lipoprotein (LDL) cholesterol and very low density lipoprotein (VLDL) cholesterol in the serum of STZ-induced diabetic rats

**Group**	**TG (mg** **/** **dl)**	**TC (mg** **/** **dl)**	**HDL-C (mg** **/** **dl)**	**LDL-C (mg** **/** **dl)**	**VLDL-C (mg** **/** **dl)**
**None-diabetic Control (C)**	31.35±2.1††	49.3±2.0†	25.04±1.3 ††	14.5±1.0 ††	5.5±0.3 ††
**Non-diabetic+ Honey (CH)**	32.84±1.5 ††	54.8±1.04 ††	24.1±2.0 ††	22.3±3.0 ††	6.7±0.3 ††
**Diabetic Control (CD)**	52.59±1.9٭	83.7±1.9٭٭	13.34±0.5 ٭٭	59.6±1.7٭٭	10.4±0.4 ٭٭
**Diabetic+ Honey (DH)**	41.48±1.1٭٭††	74.51±1.0 ٭٭††	23.12±0.8 ††	39.7±2.4 ٭٭††	8.3±0.2 ٭٭††
**Diabetic+ Metformin (DM)**	45.97±1.4 ٭٭	70.25±0.5 ٭٭††	11.64±0.08 ٭٭	49.2±0.5 ٭٭††	8.8±0.3 ٭٭††
**Diabetic+ Metformin+ Honey (D.M.H)**	40.49±0.9 ٭٭††	67.1±1.5 ٭٭††	19.93±0.72 ٭٭††	42.4±1.2 ٭٭††	8.1±0.2 ٭٭††

Data are expressed as mean±SEM. Each group consisted of five to seven animals. Values are statistically significant at:

* p<0.05,

** p<0.01 compared with non-diabetic control;

† p<0.05,

†† p<0.01 compared to diabetic control.

**Table 4:** Comparison in terms of p< 0.05 for TG, TC, LDL, HDL and VLDL. Common letters indicate non-significant difference in columns

**Group**	**TG** ** (mg/dl)**	**TC** ** (mg/dl)**	**HDL-C** ** (mg/dl)**	**LDL-C** ** (mg/dl)**	**VLDL-C** ** (mg/dl)**
**None-diabetic Control (C)**	A	A	A	A	A
**Non-diabetic+ Honey (CH)**	A	A	A	A	A
**Diabetic Control (CD)**	C	D	C	D	C
**Diabetic+ Honey (DH)**	B	C	AB	B	B
**Diabetic+ Metformin (DM)**	BC	BC	C	C	B
**Diabetic+ Metformin+ Honey (DMH)**	B	B	B	BC	B

## Discussion

STZ has a selective cytotoxic action on the β-cells in the islets of Langerhans and induces diabetes mellitus in experimental animals (Brenna et al., 2003 ▶). Honey lowers blood glucose in diabetic rats (Erejuwa et al., 2012b ▶; Erejuwa, 2011c ▶). Unlike honey, mechanism of action of Metformin is well known in lowering blood sugar (Erejuwa et al., 2011b ▶). The Hypoglycemic effect of honey can be exerted by fructose, which is one of the major Manufactures (Erejuwa et al., 2011b ▶). Fructose does not increase plasma glucose and its metabolism does not require insulin (Erejuwa et al., 2011b ▶). One of the unique features of Tualang honey compared with other types of honey is a 1:1 ratio of fructose to glucose (Erejuwa et al., 2011b ▶). Natural honey, fructose (35.56%), glucose (36.04%), fructose/glucose ratio (0.986%), which was used in this study had ratio of 1:1 for fructose to glucose, too. In addition, physicochemical studies showed antioxidant properties for Ilam natural honey (results not shown). Fructose is monosaccharide and absorbed from the gastrointestinal tract slower than glucose. Therefore, its metabolism is largely independent of insulin and quickly removed by the liver. Therefore, its consumption leads to only a slight increase in blood sugar and can be appropriate sweetener for patients with diabetes type 2 (Crapo et al., 1980 ▶). In addition, honey contains various minerals and antioxidants. Honey increases antioxidant agents such as oral intake levels of vitamin C, beta-carotene, serum uric acid, and glutathione reductase (Al-Waili et al., 2003 ▶). Increase in phenol levels in the plasma of healthy individuals is due to the presence of phenolic antioxidants in honey (Schramm et al., 2003 ▶). The amount and type of antioxidants depends largely upon the floral/variety of the honey (Rosa, 2011 ▶). The honey used in this study was prepared from Ilam forest, which has a high diversity of medicinal plants. Diversity of medicinal plants has positive influence on therapeutic and antioxidant properties of honey. The scientific name of Iranian honey bee is *Apis mellifera meda* which has a variety source of the plants.

Obesity among patients with type 2 diabetes is a common problem that affects 90 percent of these individuals (Anderson et al., 2003 ▶). However achievements of weight loss in diabetic patients seem difficult (Wing et al., 1987 ▶). On the other hand, using medications to control blood sugar is associated with weight gain. Diet weight loss is the most effective treatment for this problem, but for many patients, following these restrictive diets is difficult. Limiting consumption of sweets is one of the limitations of the patients. The sweeteners are delicious and have become more and more accepted (Mann, 1987 ▶). Antioxidants have been associated with weight loss in obesity (Oben et al., 2007 ▶; Heber et al., 2003 ▶). 

The results of this study show that natural honey or Metformin in combination with honey significantly (p<0.01) improved body weight compared with diabetic control rats. However, the diabetic control rats showed significant increases in TG, TC, VLDL, and LDL cholesterol, and decreases in HDL levels. Our findings indicate that Metformin alone did not lead to any improvement in diabetic rats (Figure 1). Similarly, some researchers reported significant lipid abnormalities in diabetic rats (Ohno et al., 2000 ▶). Administration of natural honey or natural honey with Metformin significantly (p<0.01) decreased the levels of TG and VLDL cholesterol in diabetic rats compared with diabetic control rats and diabetic rat treated with Metformin alone. Natural honey and combination of Metformin and natural honey increased the level of HDL cholesterol compared with Metformin alone in diabetic rats. Natural honey, Metformin and combination of Metformin and natural honey significantly (p<0.01) decreased the levels of TC and LDL cholesterol in diabetic rats compared with diabetic control rats. Diabetic rats that received Metformin show decreased levels of TC, VLDL, and LDL. Our finding is in agreement with Erejuwa et al. (2011b ▶) on amelioration effect of this drug. Besides, significant lower levels of TC, TG, VLDL, and LDL cholesterol and higher levels of HDL cholesterol were observed in diabetic rats which received combination of Metformin and natural honey. This observation indicates that Metformin in combination with natural honey is more effective than Metformin alone. Our data supports higher therapeutic effects on lipid abnormalities in Ilam honey than Tualang honey. For example, ANOVA differences between diabetic control (CD) and diabetic groups treated with honey (DH) is significant at p<0.01, while, the differences in Erejuwa et al. (2011b ▶) study was significant at p<0.05. Moreover, our results for TC and LDL cholesterol showed highly significant difference (p<0.01) between diabetic control (CD) and diabetic groups treated with honey (DH), but application of Tualang honey did not show any significant difference between CD and DH groups. Combination of Ilam natural honey and Metformin also had a better effect on control of lipid abnormality compared with combination of Tualang honey and Metformin (Table 3). 

Results of DPPH and FRAP assays of Ilam natural honey indicated that this honey with57.25% inhibition and 328.32 μM Fe (II) has higher antioxidant properties than Tualang honey reported with 41.3% inhibition and 322.1 μM Fe (II) (Mohamed et al., 2010 ▶). Moreover, Ilam honey has a very low hydroxy methyl furfural (HMF) (3.40 mg/kg). High levels of this factor indicate that the honey is harmful to health and counted carcinogenic (Feás et al., 2010 ▶). In conclusion, application of Ilam honey in our investigation showed prevention of weight loss in diabetic rats and had higher therapeutic effects on lipid abnormalities compared with Tualang honey used in Erejuwa et al. (2001 b ▶) study. Our results also indicated that combination of Ilam natural honey with Metformin has a better effect compared with Metformin alone in STZ-induced diabetic rats. 
